# Selected Properties of Plywood Bonded with Low-Density Polyethylene Film from Different Wood Species

**DOI:** 10.3390/polym14010051

**Published:** 2021-12-23

**Authors:** Pavlo Bekhta, Orest Chernetskyi, Iryna Kusniak, Nataliya Bekhta, Olesya Bryn

**Affiliations:** 1 Department of Wood-Based Composites, Cellulose, and Paper, Ukrainian National Forestry University, 79057 Lviv, Ukraine; kusnyak@nltu.edu.ua (I.K.); n_bekhta@nltu.edu.ua (N.B.); bryn_o@nltu.edu.ua (O.B.); 2 Shpon Shepetivka LLC, 30400 Shepetivka, Ukraine; Lokiorest@gmail.com

**Keywords:** plastic film-bonded plywood, polyethylene film, physical–mechanical properties, bonding strength, wood species

## Abstract

In this work, the effects of wood species and thickness of low-density polyethylene (LDPE) film on the properties of environmentally-friendly plywood were studied. Rotary-cut veneers from four wood species (beech, birch, hornbeam and poplar) and LDPE film of four thicknesses (50, 80, 100 and 150 µm) as an adhesive were used for making plywood samples. The findings of this study demonstrated that plywood samples using all the investigated wood species bonded with LDPE film showed satisfactory physical–mechanical properties. Poplar veneer provided the lowest values for bending strength, modulus of elasticity and thickness swelling of all the plywood samples, but the bonding strength was at the same level as birch and hornbeam veneer. Beech plywood samples had the best mechanical properties. An increase in LDPE film thickness improved the physical–mechanical properties of plastic-bonded plywood.

## 1. Introduction

**Figure 1:**
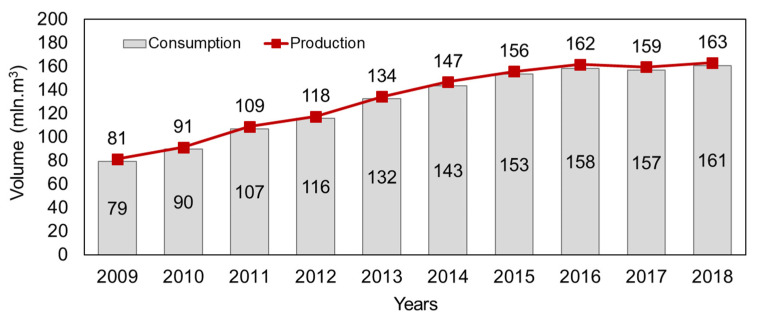
World production and consumption of plywood according to FAO statistics.

Wood-based materials are popular materials that are still used extensively as an alternative to solid wood across many industries. Among wood-based materials, plywood is widely used in diverse fields, including housing construction, vehicles, laser engraving and interior decoration, furniture production and many others [1,2]. According to the latest data released by FAO [3], during the ten-year period from 2009 to 2018, plywood global production and consumption volumes increased by 101% and 104%, respectively, which amounted to record values in 2018 of over 163 and 161 million m^3^, respectively (Figure 1). However, over the past two years, due to global problems related to the coronavirus, there has been a stagnation in plywood production and consumption. In 2019, global plywood production decreased by 0.19% in comparison with 2018 [3].

Plywood products are manufactured using different formaldehyde-based adhesives [4]. Despite the fact that these adhesives have good adhesive properties [5], the formaldehyde is emitted in the production and use of plywood. Formaldehyde is a human carcinogen [6] and poses a major risk to human health. The impact of the living environment on human health has become increasingly important, and has resulted in a change in attitudes towards issues related to the environment and human health. Therefore, the development of plywood with alternative formaldehyde-free adhesives has attracted increasing attention from both the industrial community and academia in recent years. Demand is growing for plywood that uses new formaldehyde-free adhesive compositions for wood bonding [7]. However, the use of some newly developed adhesives at an industrial scale is limited due to the high cost of modification and poor water-resistance [7].

Therefore, the application of thermoplastics and their copolymers for bonding plywood is promising [8], and formaldehyde-free plywood has been successfully produced using thermoplastic polymers as adhesives [8,9,10,11,12,13,14,15,16,17,18,19,20,21,22,23,24,25,26,27,28]. Different types of thermoplastic polymers have been used for wood veneer bonding: low-density polyethylene (LDPE) [9,10,11], high-density polyethylene (HDPE) [12,13,14,15,16,17,18,19,20,21], PS [22,23], PP [8,24,25], PVC [26], poly-b-hydroxybutyrate [27], co-polyamide and co-polyester [11]. Thermoplastic polymers are used in different forms for the bonding of wood veneer [8,11,12,13,14,15,16,17,18,19,28]. The application of thermoplastic polymers in the form of a film for bonding plywood is the most effective method and greatly simplifies the technology. Some of the advantages and disadvantages of using thermoplastic film for bonding plywood were described in our previous work [11,21]. The great advantage of these polymers is that the amount of formaldehyde emission from the plastic-bonded plywood is almost zero [28]. In addition, the use of plastic film in the production of plywood enables the production of larger-sized products at lower pressure [11,21] compared to the manufacture of conventional UF and PF-bonded plywood or wood–polymer composites (WPC) [29].

Plywood can be produced from different wood species, and the species determines the physical–mechanical properties of the plywood [11,30]. Most of the above-mentioned studies used poplar [13,14,15,17,18,22,25,28], eucalyptus [19,20,26,27], or birch [8,11] for bonding with thermoplastic polymers. There are also several works on the use of red Seraya [24], spruce [10,11], alder [21], beech [11], Masson pine [12] and Amescla wood [16]. There is limited data in the literature on the use of beech and hornbeam wood veneers. In Ukraine, birch is the most popular wood for plywood production. However, due to the limited stocks of birch raw materials, plywood producers are being forced to find other wood species to replace birch. Beech, hornbeam and poplar wood species are already being considered as alternative raw materials for plywood production.

Therefore, in this study we investigated the possibility of making plywood using beech, hornbeam and poplar, as well as birch for comparison, bonded with thermoplastic film of different thicknesses. This will expand our knowledge of the raw materials that can be used as a base for the manufacture of plastic plywood, as well as expand the applications of such plywood.

## 2. Materials and Methods

### 2.1. Materials

Rotary-cut veneers of four hardwood species of poplar (*Populus alba* L.), birch (*Betula verrucosa* Ehrh.), beech (*Fagus sylvatica* L.), and hornbeam (*Carpinus betulus* L.) with a thickness of 0.75 mm, 1.55 mm, 0.45 mm and 1.50 mm, respectively, were used in the experiments. The moisture content of the veneer sheets was about 6 ± 2%. Birch is the wood species most frequently used for the production of peeled veneer and plywood in Ukraine. Poplar, beech and hornbeam were selected as alternative wood species, all of which have significant reserves in Ukraine. Veneer sheets were obtained from a local producer of peeled veneer and plywood panels. Each veneer sheet was visually checked, and sheets without any visible defects were selected. In addition, the veneer was manufactured from one batch of raw materials. This was done to minimize the effects of wood structure and origin on the findings of the experiment.

Low-density polyethylene (LDPE) film (LLC “Planet Plastic”, Irpin, Ukraine) with the same dimensions as the veneers and thicknesses of 50 µm, 80 µm, 100 µm and 150 µm, density of 0.92 g/cm^3^ and a melting point of 105–110 °C was used for the bonding of the wood veneers.

### 2.2. Manufacturing of Plywood Samples

**Figure 2:**
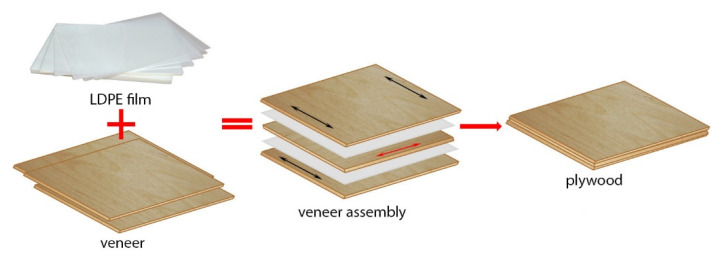
Schematic of plywood samples’ production.

Three-layer plywood samples measuring 300 mm × 300 mm were prepared. LDPE film, which mainly works as the adhesive connecting between two adjacent veneers, was used to manufacture the plywood samples. One sheet of film was incorporated between the veneer sheets. The prepared veneer assemblies (Figure 2) were subjected to hot pressing in the lab press at a pressure of 1.4 MPa and temperature of 160 °C for 4.5 min (Table 1). The mass per unit area of LDPE film at thicknesses of 50 µm, 80 µm, 100 µm and 150 µm was approximately 46.0, 73.6, 92.0 and 138 g/m^2^, respectively.

After the plywood samples were removed from the hot press, they were subjected to the cold pressing stage at room temperature. This was performed to release internal stresses and reduce the warping of samples. Three plywood samples were prepared at each condition.

### 2.3. Testing the Plywood Panels

After bonding, the plywood panels were air conditioned at 20 ± 2 °C and 65 ± 5% (RH). The physical properties (density, water absorption (WA) and thickness swelling (TS)) and mechanical properties (bending strength (MOR), modulus of elasticity in bending (MOE) and shear strength) of the LDPE film-bonded plywood samples were determined according to the standards [31,32,33,34,35]. The shear strength was measured after pre-treatment for bonding class 1—dry conditions—plywood test pieces were immersed in water at 20 ± 3 °C for 24 h [33,34]. To determine the WA and TS, the samples were immersed in distilled water for 2 and 24 h. For each variant, at least ten samples were used for the shear strength test and six samples were used to determine MOR, MOE, WA and TS.

**Figure 3:**
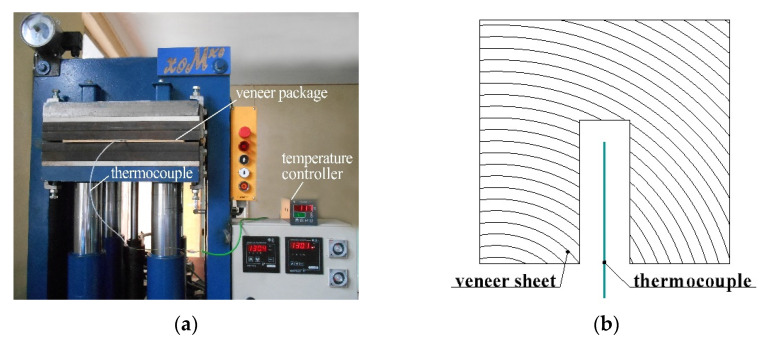
Measuring the core temperature during pressing of plywood sample (**a**) and positioning the thermocouple in the middle sheet of the plywood (**b**).

Moreover, the measurement of the core temperature inside the veneer package under given wood species and thickness of LDPE film was undertaken (Figure 3) according to the procedure described in our previous work [36].

### 2.4. Statistical Analysis

To evaluate the effect of the LDPE film thickness and wood species on the properties of plywood samples, ANOVA analysis was performed using SPSS software program version 22 (IBM Corp., Armonk, NY, USA). The effects were not considered to be statistically significant when the *p*-value was higher than 0.05 at the 95% confidence level. Duncan’s multiple range test was used to determine the significant differences between and among the groups.

## 3. Results

### 3.1. Core Layer Temperature Analysis

The melting point of the investigated film was 105–110 °C. However, the plywood samples were pressed at a higher temperature of 160 °C to ensure good fluidity of the molten LDPE film and its good penetration into the cavities of the wood [11,21]. To determine the process for heating the veneer package at a given temperature, as well as the melting and spreading of the LDPE film during pressing, the core layer temperature inside the package was measured for each film thickness and wood species.

**Figure 4:**
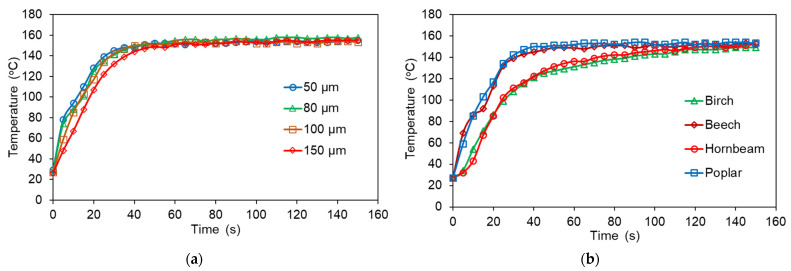
Core temperature curves of plywood samples made with (**a**) poplar veneers and different thicknesses of LDPE film, and (**b**) veneers of different wood species and LDPE film of 100 µm thickness.

The core temperature distribution inside the veneer package during pressing of the plywood panels with different film thicknesses and various wood species is shown in Figure 4. Among the investigated wood species, the samples with of poplar and beech veneers warmed up the fastest (in approximately 17 s) to the melting temperature of LDPE film (110 °C), while the samples with birch and hornbeam veneer warmed up slowly (in approximately 30 s). This can be mainly explained by the influence of the veneer thickness. The beech and poplar veneer were the thinnest, and therefore the samples using these veneers heat up faster. Analysis (ANOVA) also showed that in addition to the thickness of the veneer, the wood species affects the temperature distribution inside the sample, especially at the stage of heating to the melting point of the polymer. When the temperature inside the veneer package reaches about 150 °C (approximately after 100 s of heating), the heating curves coincide for different wood species. The effect of the thickness of the thermoplastic film on the temperature distribution inside the veneer package was insignificant (*p* > 0.05) due to its slight thickness (Figure 4a). The main factors that determine the heating rate of the veneer package with thermoplastic film are the thickness of the veneer and the wood species at the same pressing conditions.

To improve the bonding properties of plywood, the plastic must penetrate the wood before it is cured [17]. From Figure 4a,b, it can be seen clearly that a temperature of 160 °C and 4.5 min of pressing are sufficient to heat the veneer package, melt the plastic film, allow it to spread on the surface of the veneer and penetrate into the wood before it is cured. Better penetration creates stronger bonds in the sample. A study by Goto et al. [24] also showed that good melting and the penetration of polypropylene into various wooden elements and veneer spaces, generally promoted bonding. Therefore, since wood is a porous material, the most probable gluing mechanism is mechanical locking [8,13,18,19,24,37].

### 3.2. Density of Plywood Samples

The ANOVA analysis showed the significance of the effect of the wood species and LDPE film thickness on the density of plastic-bonded plywood samples. The greatest influence on the density of plywood samples was the wood species (*F* = 1610.956), while the thickness of the plastic film affects the density to a lesser extent (*F* = 23.939). Based on Duncan’s test, it was found that the values for the density of the samples made with beech, birch, hornbeam or poplar veneers differed significantly (*p* ≤ 0.05). Duncan’s test also confirmed that there was a statistically significant difference in the density values of plywood samples bonded by film with a thickness of 50, 80, 100 or 150 µm (*p* ≤ 0.05). Therefore, the density of the LDPE film should be taken into account in the manufacture of plywood panels. However, the differences in the density made with LDPE film of 50, 80 and 100 µm were insignificant (*p* > 0.05). Similar results were obtained by other authors [30,38] who found that thermosetting adhesives had a significant effect on the density of laminated materials.

**Figure 5:**
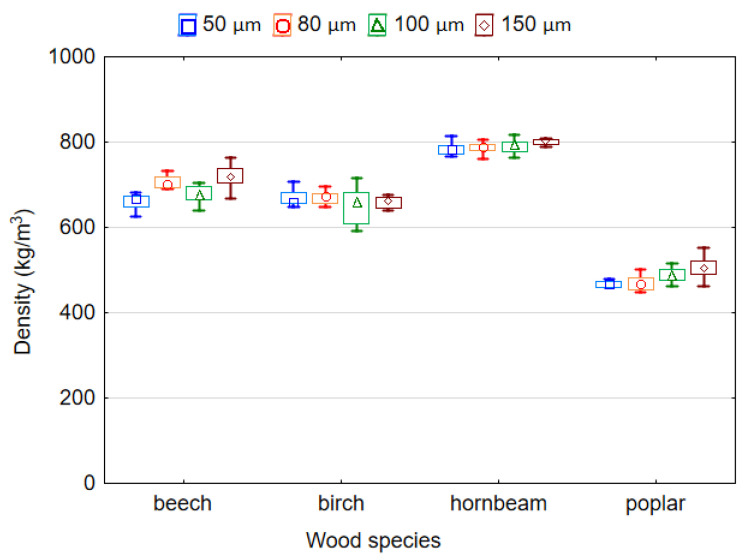
Density of plywood bonded by LDPE film.

It was found that the density of plywood samples was significantly affected by the density of the veneers. Naturally, the smallest and highest density values correspond to the samples with poplar (481.8 kg/m^3^) and hornbeam (790.8 kg/m^3^) veneers, respectively (Figure 5). This is due to the higher density of hornbeam (730 kg/m^3^) in comparison with poplar (390 kg/m^3^) veneers.

### 3.3. Bending Strength and Modulus of Elasticity of Plywood Samples

**Figure 6:**
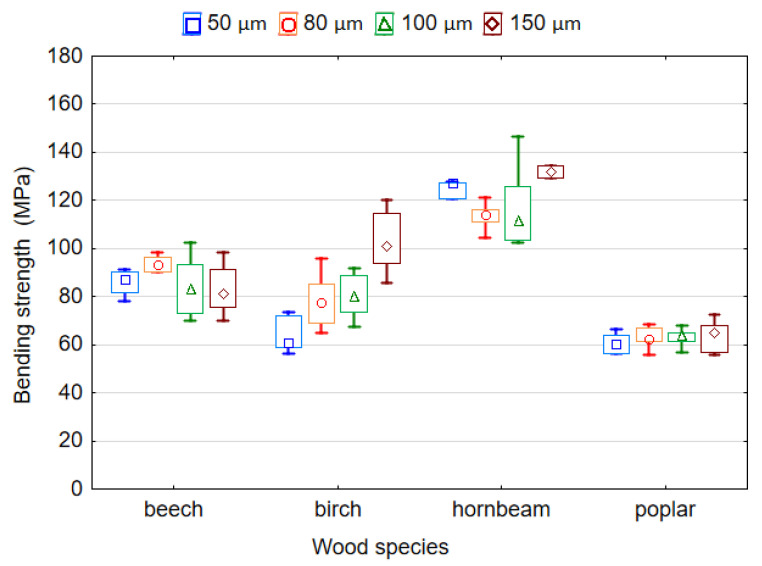
Bending strength of plywood bonded by LDPE film.

**Figure 7:**
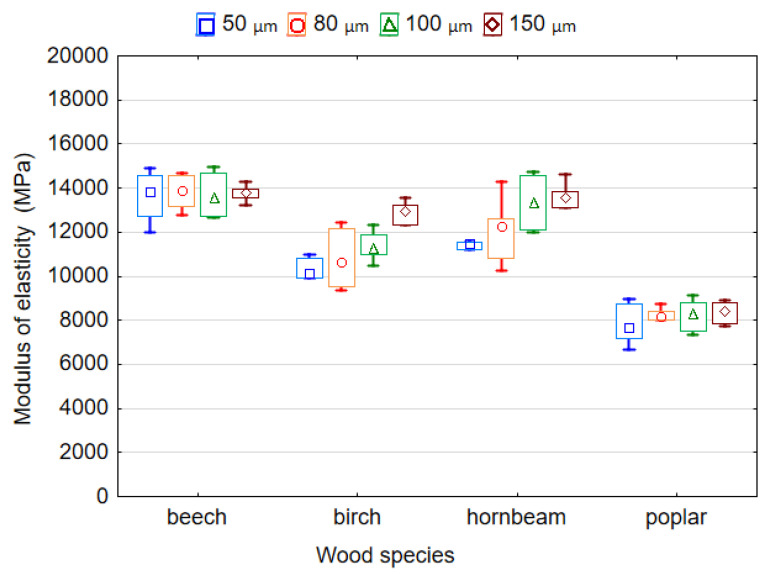
Modulus of elasticity of plywood bonded by LDPE film.

The effect of the thickness of LDPE film and wood species on the MOR and MOE of plywood samples is shown in Figure 6 and Figure 7. It was found that both variables significantly affect the MOR and MOE (*p* < 0.05). The ANOVA analysis showed that according to the *F-*values, the wood species has the greatest effect on the MOR (*F* = 122.307) and MOE (*F* = 108.160) of the plywood samples, while the thickness of the plastic film affected the MOR (*F* = 7.488) and MOE (*F* = 5.731) to a lesser extent. The values of MOR for samples made using film with thicknesses 50, 80 and 100 µm differed insignificantly (*p* > 0.05). Similarly, as for MOR, the difference in the values of MOE for the thicknesses of 80 and 100 µm as well as 100 and 150 µm was also insignificant (*p* > 0.05).

The highest MOR and MOE values of 94.8 MPa and 11,750.0 MPa, respectively, corresponded to samples bonded by LDPE film with a thickness of 150 µm. The smallest MOR and MOE values of 82.6 MPa and 10,490.0 MPa, respectively, corresponded to the samples bonded by LDPE film with a thickness of 50 µm. This was predictable given that previous studies have shown that the properties of plywood bonded by plastic may decrease with low amounts of adhesive [27]. Moreover, it should be noted that both the MOR and MOE of plywood samples increased with an increase in the thickness of the film from 50 to 150 µm. This can be explained by the fact that the consumption of polymer increases with increases in the film thickness, which leads to better and more complete filling of the wood cavities. In turn, this leads to the formation of more adhesive locks. Goto et al. [24] showed that penetration of thermoplastic polymer into various wooden elements and veneer spaces, mainly promoted bonding. Many authors point out that since wood is a porous material, the most probable mechanism for bonding of thermoplastic polymers to wood is mechanical locking [8,13,18,19,24,37].

It is to be expected that the wood species affects the MOR and MOE of plywood samples differently. The highest MOR and MOE values of 120.4 MPa and 13,746.1 MPa, respectively, correspond to the samples made using hornbeam and beech, respectively. The lowest MOR and MOE values of 62.5 MPa and 8156.2 MPa, respectively, correspond to the samples made using poplar veneer. Thus, because hornbeam and beech wood have a higher density and better strength properties than poplar wood, they provide higher MOR and MOE values. The mean values for the MOR of LDPE-bonded plywood samples were: 82.96–93.57 MPa for beech veneer; 63.70–102.65 MPa for birch veneer; 113.55–129.45 MPa for hornbeam veneer; and 60.47–63.80 MPa for poplar veneer. The bending strength of beech, birch, hornbeam and poplar solid woods are 104, 110, 128 and 68 MPa, respectively [39]. Therefore, it was found that the plywood samples had MOR values similar to those of the solid wood. In another study [40], the statistically significant effects of veneer wood species on some properties of LVL were also noted.

It has been well established [39] that there is a linear relationship between MOR and density, that is, the MOR increases with increasing density. However, based on the results presented in Figure 6, we can conclude that the relationship between MOR and the density of plywood samples is more complex. That is, the MOR of the sample depends not only on its density, but also on the thickness of the veneer used, the amount of applied polymer, the thickness of the plywood samples, etc.

This explains why the samples of birch and poplar plywood with a minimum LDPE film thickness of 50 μm had almost the same bending strength. In this case, not only the densities of the veneer and plywood, but also the thicknesses of the veneer and plywood influenced the bending strength. The thickness of the film at 50 μm can be neglected. These parameters were smaller for poplar plywood compared to birch plywood. The same applies to beech and birch plywood. Beech veneer had a lower density (605 kg/m^3^) and less thickness compared to birch veneer (655 kg/m^3^). Beech plywood, which had a lower density and thickness, showed greater bending strength than birch plywood, which had a higher density and thickness.

This is confirmed by previous studies [41,42,43] that showed that plywood samples made using thin veneer had a higher bending strength compared to samples made using thick veneer. The decrease in strength may be due to the fact that the thicker the veneer, the deeper and the longer the lathe checks will be [1,43]. In addition, there is a linear relationship between MOR and the thickness of plywood samples [41]. MOR decreases with an increase in the thickness of the samples [41,44].

### 3.4. Shear Strength of Plywood Samples

**Figure 8:**
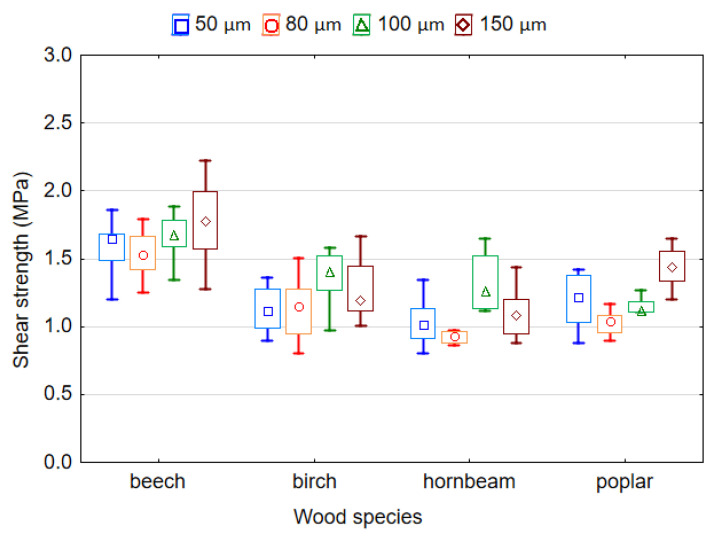
Shear strength of plywood bonded by LDPE film.

Shear strength is one of the most important mechanical properties of plywood. Figure 8 shows the effects of the wood species and thickness of LDPE film on the shear strength of plywood samples. The obtained mean values of shear strength ranged from 1.54 to 1.77 MPa for beech veneer, from 1.13 to 1.36 MPa for birch veneer, from 0.94 to 1.31 MPa for hornbeam veneer and from 1.03 to 1.44 MPa for poplar veneer. All shear strength values met the requirements of EN 314-2 standards [34] for Class 1 (dry conditions) plywood. However, hornbeam samples bonded with 80 µm thickness film failed (0.94 MPa). Among the studied wood species, the highest shear strength mean values correspond to the plywood samples made with birch and beech veneers, that is, 1.22 and 1.64 MPa, respectively. The shear strength mean values of the samples from hornbeam and poplar veneer were the smallest at 1.09 and 1.19 MPa, respectively. The shear strength values for the samples made with birch and poplar veneers differ insignificantly based on Duncan’s test (*p* > 0.05).

The results obtained in this study contradict the results of other researchers who have indicated that wood species with high density have higher shear strength values [30,45]. It is known that the formation of bonding strength largely depends on the amount of plastic film, its penetration of the wood cavities, and the formation of mechanical locks [46]. Apparently, the similar values of shear strength in the plywood samples made from poplar, birch and hornbeam veneers can be explained by the different thickness and porosity of the wood veneer used. Given the same film thickness, the amount of polymer that will penetrate per unit volume of poplar veneer (veneer density 390 kg/m^3^ and thickness 0.75 mm) will be much greater than the amount of polymer that will penetrate per unit volume of birch (veneer density 655 kg/m^3^ and thickness 1.55 mm) or hornbeam veneers (veneer density 730 kg/m^3^ and thickness 1.50 mm). In addition, less polymer will penetrate the cavities of birch and hornbeam veneers because their porosity is lower than poplar veneer. It is well known that better and deeper penetration of polymers into wood creates stronger bonds in the samples [8,13,18,19,21,24,37].

In addition, previous studies have shown that not only the density but also the thickness of the veneer also significantly affects the bonding strength [41,43]. They found that increasing veneer thickness usually leads to a decrease in bonding strength. Therefore, as we can see from Figure 8, plywood made of poplar veneer of lower density and thickness shows the similar bonding strength as plywood made of birch and hornbeam veneer, which have higher density and thickness.

The lowest shear strength values of 1.19 MPa and 1.24 MPa correspond to the samples bonded by LDPE film with a thickness of 80 and 50 µm, respectively, while the highest shear strength values of 1.36 MPa and 1.37 MPa correspond to the samples bonded by LDPE film of 100 and 150 µm, respectively. However, based on Duncan’s test the values of shear strength in the samples bonded by LDPE film with a thickness of 80 and 50 µm, as well as 100 and 150 µm, differ insignificantly (*p* > 0.05).

The changes in shear strength might be related to the penetration of the LDPE polymer in the wood. Wood is a porous material, and thus penetration of adhesive plays an important role in wood adhesion. The adhesive must penetrate the wood before it is cured to provide adequate mechanical interlocking [17]. The lowest shear strength in the plywood samples bonded with LDPE film of 50 and 80 µm is due to the low adhesive spread rate for these thicknesses, which was equivalent to 46.0 and 73.6 g/m^2^, respectively. This is a lower amount of adhesive than the amount used in practice for liquid thermosetting adhesives. The molten LDPE film was pressed into the vessels and cracks of the veneer during the pressing process. Therefore, when less molten film remains between the sheets of the veneer, this causes a reduction in the bonding strength [11,21]. Increasing the thickness of the film, and hence the amount of polymer, leads to increased bonding strength. Therefore, it is recommended that LDPE films with a thickness of 100 µm and more be used. An obvious increase in the bonding strength with an increase in the number of HDPE layers was observed by Chang et al. [17].

### 3.5. Water Absorption and Thickness Swelling of Plywood Samples

**Figure 9:**
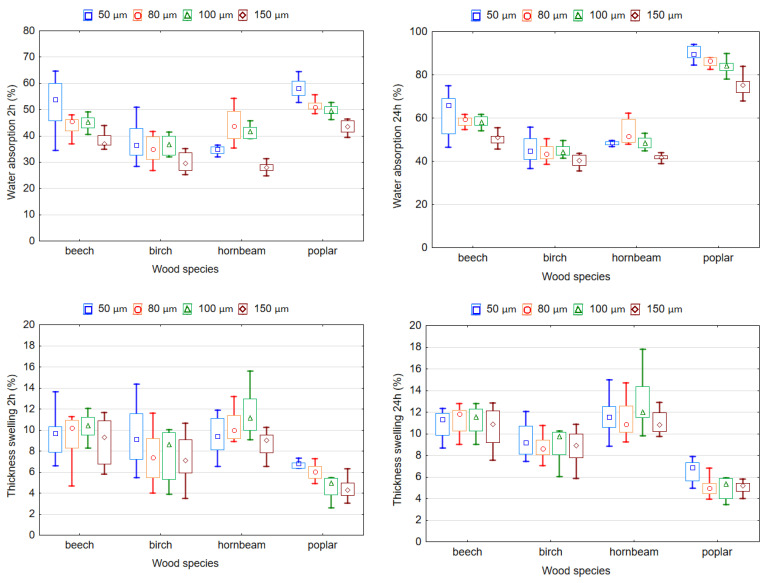
Water absorption and thickness swelling of plywood bonded by LDPE film.

Figure 9 shows the influence of the wood species and thickness of the LDPE film adhesive on the WA and TS of plywood samples. The WA and TS increases with an increase in the soaking time. The smallest values of WA and TS were observed during soaking for 2 h, and then the WA and TS increased. ANOVA analysis showed that both variables significantly (*p* < 0.05) affect WA and TS. According to the *F* values, the wood species had the greatest effect on the WA (2 h) and TS (2 h) (*F* = 127.757 and *F* = 50.127, respectively) and WA (24 h) and TS (24 h) (*F* = 883.623 and *F* = 162.324, respectively) of the plywood samples, while the thickness of the plastic film affected the WA (2 h) and TS (2 h) (*F* = 54.642 and *F* = 3.953, respectively) and WA (24 h) and TS (24 h) (*F* = 53.434 and *F* = 2.941, respectively) to a lesser extent.

The highest WA values after 2 and 24 h of soaking in water were found in the plywood samples from beech and poplar veneers, while the lowest corresponded to the hornbeam and birch veneers. The lowest value of WA (24 h) was recorded in samples made with birch veneer (43.7%), with an increase in samples made with hornbeam (48.0%) and beech (57.0%) veneers, and the highest was recorded in samples made with poplar veneer (84.0%). A different trend was observed for TS (24 h). The lowest TS (24 h) values corresponded to the samples made with poplar veneer (5.4%), while the highest TS (24 h) corresponded to the samples made with birch, beech or hornbeam veneers (9.0%, 10.9% and 11.7%, respectively). The values of TS (24 h) for birch and beech veneer do not differ significantly (*p* > 0.05).

The obtained results are explained by the differences in the anatomical structure of the wood that was used. It can be seen from Figure 9 that the WA of plywood samples decreases with an increase in the density of the wood veneers. This is because the number of pores (internal voids) in the samples with higher density is less than in the samples with lower density. Therefore, the WA of plywood samples from high-density wood veneers occurs mainly due to the penetration of moisture into the cell walls, and this process is much slower than filling the pores with moisture [39]. Plywood samples from poplar veneers had the lowest percentage of TS because more polymer penetrated per unit volume of this veneer than per unit volume of birch and hornbeam veneers. The obtained values of WA are in good agreement with the commonly accepted statement that WA is related to density [47]. The higher density results in a lower number of empty spaces, and consequently, lower WA.

Plywood samples produced with a higher LDPE film thickness showed lower percentages of WA and TS compared to the plywood samples produced with a lower film thickness. The lowest TS and WA values correspond to the samples bonded with LDPE film with a 150 µm thickness. The LDPE film with a 50 µm thickness showed the worst TS and WA due to the low consumption of such a film thickness. This causes insufficient filling of wood cavities with molten polymer. The amount of adhesive has been found to have a similar effect on the TS and WA of wood materials such as particleboard or medium-density fiberboard [48]. Increasing the amount of adhesive prevents the penetration of water into the panels and thus reduces TS and WA. The values of WA (24 h) in the samples bonded with a film thickness of 50 and 80 µm differ insignificantly (*p* > 0.05). The differences between the values of TS (24 h) in the samples bonded with a film thickness of 50, 80 and 100 µm as well as 80, 100 and 150 µm were insignificant (*p* > 0.05).

## 4. Conclusions

LDPE film of different thicknesses (50, 80, 100 and 150 µm) was successfully used for the bonding of plywood using veneer from various wood species—beech, birch, hornbeam and poplar. The wood species significantly affects the physical and mechanical properties of plywood. High-density beech, birch and hornbeam wood veneers provide the plywood with higher density, MOR, MOE and TS but lower WA than low-density poplar veneer. By contrast, plywood samples made using poplar veneer showed bonding strengths identical to samples made using birch and hornbeam veneers. This is due to not only the effect of wood density, but also the thickness of the veneer and the amount of polymer that penetrates into the wood. The plywood samples manufactured with thin veneers had better bonding strength than those manufactured with thick veneers. The beech plywood samples had the highest mechanical properties values. The properties of the plywood samples increased with an increase in the thickness of the LDPE film from 50 to 150 µm. A thin veneer package heats up faster than a thick veneer package. The thickness of the plastic film, due to its small value, did not affect the heating rate of the veneer package. Environmentally-friendly thermoplastic-bonded plywood panels can be used successfully in indoor applications.

## Figures and Tables

**Table 1:** Manufacturing conditions of plywood samples.

Adhesive	Wood Species	Thickness of Film (µm)	Pressing Temperature (°C)	Pressing Pressure (MPa)	Pressing Time (min)
LDPE film	beech, birch, hornbeam, poplar	50, 80, 100, 150	160	1.4	4.5

## Data Availability

The data that support the findings of this study are available upon reasonable request from the authors.
